# Case Report: Henoch–Schönlein-like purpura as an unusual presentation of acute hepatitis E virus infection

**DOI:** 10.3389/fimmu.2026.1937756

**Published:** 2026-10-08

**Authors:** Veronica Moronti, Verdiana Zulian, Silvia Pauciullo, Martina De Sanctis, Angela Bibbò, Daniela Pietrobono, Simona Talarico, Andreina Gubbiotti, Massimo Scorretti, Luca Moriconi, Anna Rosa Garbuglia

**Affiliations:** 1 Unità Operativa Complessa (U.O.C.) Internal Medicine, San Camillo de Lellis Hospital, Rieti, Italy; 2 Laboratory of Virology and Laboratories of Biosafety, National Institute for Infectious Diseases “Lazzaro Spallanzani”-IRCCS, Rome, Italy; 3 Unità Operativa Complessa (U.O.C.) Clinical Pathology, San Camillo Forlanini, Rome, Italy

**Keywords:** acute hepatitis, clinical case, extrahepatic manifestations, hepatitis E virus, palpable purpura

## Abstract

**Introduction:**

Hepatitis E virus (HEV) infection is increasingly recognized as a cause of extrahepatic manifestations; however, cutaneous purpuric manifestations are exceptionally rare, with only one previous case of Henoch–Schönlein purpura (HSP) reported in association with acute HEV infection.

**Methods:**

This study describes the case of a 91-year-old man admitted with palpable purpura of the lower limbs, associated with jaundice, hypertransaminasemia, and cholestatic abnormalities. Molecular and serological assays were performed.

**Results:**

Serological testing showed anti-HEV IgM and IgG positivity, and HEV RNA was detected in plasma at a viral load of 15,002 IU/mL. The patient reported consumption of wild-boar meat in the month preceding symptom onset. The purpura resolved spontaneously approximately 10 days after onset, and liver parameters progressively improved, with HEV RNA becoming undetectable at follow-up. Phylogenetic analysis of the patient-derived sequence identified the strain as HEV genotype 3f, belonging to the 3f2 subcluster. The patient-derived sequence showed similarity with European HEV-3f/3f2 human strains and with swine and wild boar strains, supporting a likely zoonotic food-borne origin. Plasma interleukin-6 (IL-6) was elevated at admission during the purpuric manifestation.

**Discussion:**

To our knowledge, this is the first Italian report of an HSP-like purpura temporally associated with acute HEV genotype 3f infection. This case highlights that acute HEV infection may be considered in the differential diagnosis of patients presenting with palpable purpura, while emphasizing the exceptional rarity of this manifestation.

## 1 Introduction

Hepatitis E virus (HEV) is considered an emerging viral pathogen and represents one of the leading causes of acute viral hepatitis worldwide. According to global estimates, HEV caused approximately 20 million infections and about 3,450 to 70,000 deaths each year (1, 2).

Phylogenetically, HEV is classified into eight genotypes. Genotypes (GTs) 1 and 2 exclusively infect humans and are transmitted mainly through the fecal–oral route, particularly via contaminated water in areas with poor sanitation. In contrast, GT3 and GT4 are zoonotic and can be transmitted to humans through the food chain, particularly via the consumption of undercooked pork products (3). These genotypes have been detected in several animal reservoirs, especially pigs and wild boar, but also in deer and rabbits (4, 5). GT3 predominates in industrialized countries and is mainly responsible for sporadic cases and small outbreaks of hepatitis E (6, 7). GT5 and GT6 have been isolated in wild boars, with no human infections reported to date. GT7 and GT8 have been identified in camels (8, 9); notably, GT7 infection has been described in humans following consumption of camel milk and meat (8, 9). Most acute HEV infections are asymptomatic or self-limiting, and symptomatic icteric hepatitis is less common. However, in severe cases, acute liver failure may occur, with mortality rates reaching up to 30% among pregnant women (10). Furthermore, older adults and patients with pre-existing liver disease are at increased risk of severe acute hepatitis E and adverse clinical outcomes (11, 12), whereas chronic HEV infection occurs primarily in immunosuppressed individuals (13, 14).

Over the last two decades, several extrahepatic manifestations associated with HEV infection have been reported. Some of these conditions have been described particularly in patients with chronic HEV infection. Neurological complications are among the best documented and include Guillain–Barré syndrome, neuralgic amyotrophy, myelitis, and encephalitis (15–17). Notably, HEV RNA has been detected in the cerebrospinal fluid of patients presenting with neurological symptoms, with evidence suggesting compartmentalization of HEV quasispecies within the central nervous system (18).

Acute pancreatitis has also been described as a rare extrahepatic manifestation of HEV infection. The first documented case of HEV-associated acute pancreatitis was reported in 1999 (19), and additional cases have subsequently been described in patients with acute HEV infection, mainly involving genotypes 1 and 3 (20).

Renal manifestations represent another relevant extrahepatic presentation of HEV infection. As observed for neurological complications, renal disorders have been reported in individuals infected with both GT1 and GT3 (21, 22). In addition, anti-HEV seropositivity has been described in patients with cryoglobulinemia, which may occur during active infection and has also been reported after viral clearance (23, 24).

Furthermore, several hematological disorders have been associated with HEV infection, including hemolytic anemia, particularly in patients with glucose-6-phosphate dehydrogenase deficiency, aplastic anemia, thrombocytopenia, and immune-mediated cytopenias.

Cutaneous manifestations have been less frequently reported; a single case of Henoch–Schönlein purpura (HSP) was described in a 6-year-old girl from India (25), and isolated cases of rash have also been reported (26).

In this report, we describe a case of HSP-like purpura occurring during acute hepatitis E in a 91-year-old man infected with HEV genotype 3f. To our knowledge, this is the first case reported in Italy of HSP-like purpura associated with acute HEV genotype 3f infection.

## 2 Case description

A 91-year-old man was admitted to the Internal Medicine unit of San Camillo de Lellis Hospital in Rieti with a 6-day history of HSP-like purpura of the lower limbs, associated with hypertransaminasemia and elevated cholestatic indices detected in laboratory tests performed in a private laboratory. The patient did not complain of fever, respiratory symptoms, arthralgia or arthritis, abdominal pain, diarrhea, bleeding, or abnormal urination. He did not consume alcohol or use tobacco.

He denied traveling abroad or eating uncooked foods, home-grown foods, or inadequately washed vegetables. He reported eating lunch at a restaurant daily, with two episodes of consumption of wild-boar meat in the month preceding the appearance of the purpura, one at a restaurant and one at home, as a gift from a friend. He denied having been in contact with anyone exhibiting the same symptoms.

His medical history included type 2 diabetes (T2DM), hypertensive heart disease, atrial fibrillation (AF), a permanent pacemaker for complete atrioventricular block, renal cysts, hepatic steatosis, and biliary sludge. His long-term medications before admission were bisoprolol 2.5 mg twice daily, linagliptin 5 mg once daily, rivaroxaban 15 mg once daily, and gliclazide 30 mg once daily. During hospitalization, rivaroxaban was temporarily discontinued until the international normalized ratio (INR) values improved. None of these medications had been recently introduced, and no recent dosage modifications had been made.

At admission, the patient presented with jaundice, elevated serum transaminase levels, and cholestatic indices. In the emergency room, the blood tests showed the following: total bilirubin 2.77 mg/dL (normal range 0-1.2 mg/dL), direct bilirubin 1.96 mg/dL, alkaline phosphatase (ALP) 251 U/L (normal range 35–104 U/L); gamma-glutamyl transpeptidase (GGT) 153 U/L (normal range 0–38 U/L), aspartate aminotransferase (AST) 709 U/L (normal range 10–35 U/L), alanine aminotransferase (ALT) 550 U/L (normal range 10–35 U/L), lactate dehydrogenase (LDH) 341 U/L (normal range 135–235 U/L), C-reactive protein 3.9 mg/dL (normal range <0.5 mg/dL), INR 3.06, and D-dimer 3.49 µg/mL (normal range 0-0.5 µg/mL). Serum creatinine was 1.29 mg/dL (normal range 0.51–1.17 mg/dL), and the estimated glomerular filtration rate (eGFR), calculated using the Chronic Kidney Disease Epidemiology Collaboration (CKD-EPI) equation, was 47.8 mL/min/1.73 m². Serial platelet counts, hemoglobin levels, INR, and partial thromboplastin time (PTT) values are reported in **Supplementary Table 1**.

On the first day of hospitalization, a routine urine analysis was performed and showed no glucosuria or ketonuria; proteinuria was 15 mg/dL (normal range <15 mg/dL), and the urinary albumin-to-creatinine ratio (UACR) was 80 mg/gCr (normal range <30 mg/gCr). Hemoglobin and bilirubin were absent, whereas urobilinogen was elevated at 2 mg/dL (normal range <1 mg/dL). Urine pH was 5.5, nitrites were absent, and the urine was dark yellow and clear, with a specific gravity of 1.034 (normal range 1.005–1.035). Urine sediment analysis showed 2 red blood cells/µL (normal range 0–15/µL), 0 white blood cells/µL (normal range 0–20/µL), and 1 squamous epithelial cell/µL (normal range 0–20/µL).

Blood cultures showed no bacterial growth. Serological testing for hepatitis A virus (HAV) IgM, hepatitis B surface antigen (HBsAg), antibodies against hepatitis B surface and core antigens (HBsAb and HBcAb), hepatitis C virus antibodies, and anti-parvovirus B19 IgG/IgM was negative.

HAV IgG and cytomegalovirus (CMV) IgG were positive. Anti-HEV IgM and IgG were also positive.

Epstein–Barr virus (EBV) serology performed on the second day of hospitalization showed an equivocal viral capsid antigen (VCA) IgM result (38 U/mL, gray zone 20–40 U/mL) and a positive VCA IgG result (718 U/mL, positive results >20 U/mL); the VCA IgM result was considered likely due to nonspecific cross-reactivity rather than acute EBV infection.

An immunological work-up was performed on the second day of hospitalization. Serum IgA levels were elevated at 652 mg/dL (normal range 70–400 mg/dL). Serum IgM levels were within the normal range at 176 mg/dL (normal range 40–320 mg/dL). On serum protein electrophoresis, a monoclonal component/band was detected in the gamma fraction and subsequently characterized by serum immunofixation as an IgG lambda monoclonal protein (1,881 mg/dL; reference range: 700–1600 mg/dL). Given the patient’s advanced age (91 years old), a diagnosis of monoclonal gammopathy of undetermined significance (MGUS) may be considered. Serum C3 and C4 levels were 74 mg/dL (normal range 4–500 mg/dL) and 11 mg/dL (normal range 10–40 mg/dL), respectively, and cryoglobulins were absent.

Testing for antimitochondrial antibodies (AMA), anti-smooth muscle antibodies (ASMA), anti-liver-kidney microsomal antibodies, and antineutrophil cytoplasmic antibodies (ANCA) was negative. Antinuclear antibodies (ANA) were moderately positive (titer 1:320) when tested using the ANA Hep-2000^®^ Fluorescent Test System (Immuno Concepts, Sacramento, CA, USA), but were negative using immunoblotting ANA 19 antigens and liver profile produced by ALPHADIA (Mont-Saint-Guibert, Belgium).

The extractable nuclear antigen (ENA) screening result was 8.3 chemiluminescence units (CU; normal value <20 CU), whereas anti-myeloperoxidase and anti-proteinase 3 ANCA levels were 1.4 CU and 1.5 CU, respectively (normal value for both, <20 CU).

The biopsy was performed on an active purpuric lesion on the fifth day after the onset of the eruption. Microscopic examination of the skin biopsy specimen revealed skin and subcutaneous tissue with diffuse epidermal atrophy and a mild superficial perivascular lymphocytic inflammatory infiltrate. Direct immunofluorescence for vascular IgA and C3 deposition was not performed.

The purpura resolved spontaneously approximately 10 days after its onset. Abdominal ultrasonography with liver elastography showed an enlarged liver, with irregular margins and a finely inhomogeneous echotexture due to chronic liver disease; no hypertrophy of the caudate lobe was observed. The liver parenchyma appeared slightly hyperechogenic, due to mild steatosis. Liver stiffness was 24.75 kPa, with an interquartile range-to-median ratio of 13%, a value within the cirrhotic range. The portal vein was at the upper limit of normal caliber and showed bidirectional flow; mild enlargement of the hepatic veins and the inferior vena cava was also observed. The gallbladder was filled with lithiatic concretions; the portal branches and bile ducts were of normal caliber. The spleen was normally formed.

Peak AST and ALT levels of 886 U/L and 634 U/L, respectively, were observed on day 9 after the purpura onset. ALP and GGT peaked at 292 U/L and 210 U/L, respectively, on day 11, whereas total and direct bilirubin peaked at 12 mg/dL and 9.48 mg/dL, respectively, on day 17. The platelet count decreased to 104 × 10^9^/L on day 9 and returned to the normal range by day 20. One month after purpura onset, total bilirubin decreased to 3.23 mg/dL, direct bilirubin to 2.44 mg/dL, ALP to 136 U/L, and GGT to 85.6 U/L, with normalization of transaminase levels. Three months after admission, normalization of ALP levels and reductions in total bilirubin to 1.44 mg/dL, direct bilirubin to 0.94 mg/dL, and GGT to 76.5 U/L were observed. At the sixth month after admission, total bilirubin had normalized, with GGT only slightly altered (57 U/L, normal range 0–55 U/L). The albumin level improved after the patient returned home; it was 3.41 g/dL at admission, 3.97 g/dL three months after the onset of symptoms, and 4.08 g/dL six months after (**Figure 1**).

**Figure 1:**
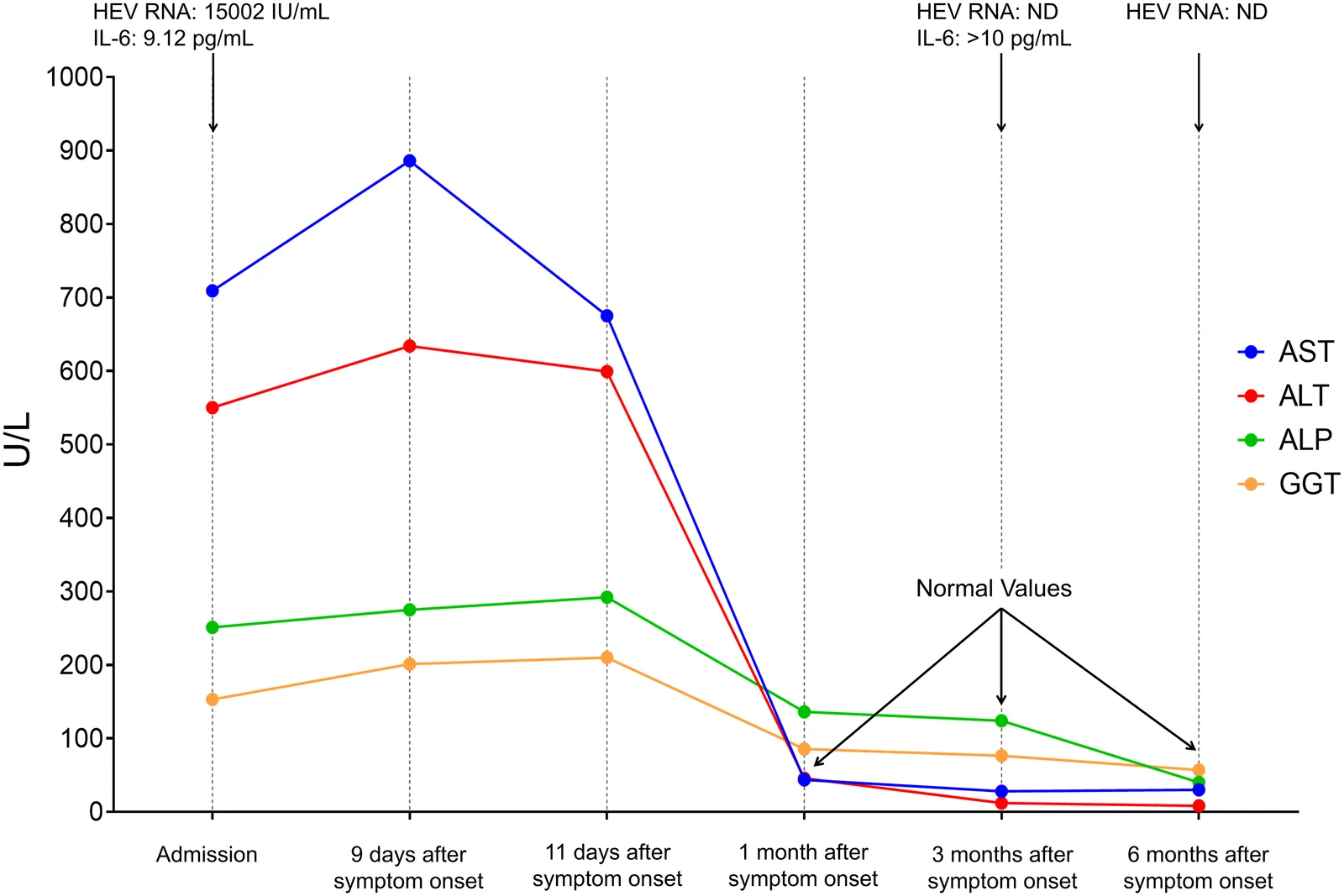
Trend of laboratory parameters. Time course of liver enzymes and cholestatic indices from hospital admission to six months after symptom onset in a 91-year-old man admitted to the Internal Medicine Unit of San Camillo de Lellis Hospital, Rieti. (AST, aspartate aminotransferase; ALT, alanine aminotransferase; ALP, alkaline phosphatase; GGT, gamma-glutamyl transpeptidase; HEV, hepatitis E virus; IL-6, interleukin-6; ND, not detected).

## 3 Materials and methods

Anti-HEV IgM and IgG were tested using a commercial chemiluminescence assay (DiaSorin, Saluggia, VC, Italy) according to the manufacturer’s instructions. HEV RNA was detected and quantified in plasma samples using the RealStar HEV RT-PCR Kit 2.0 (Altona, Hamburg, Germany) according to the manufacturer’s recommendations.

Interleukin-6 (IL-6) levels were measured using a sandwich enzyme-linked immunosorbent assay (ELISA) with the Quantikine^®^ HS ELISA Human IL-6 Immunoassay Kit (Bio-Techne/R&D Systems, Minneapolis, MN, USA), according to the manufacturer’s instructions (assay range: 0.156–10 pg/mL). ELISA plates were read using an Infinite^®^ 200 PRO M PLEX spectrophotometer (Tecan, Männedorf, Switzerland), with Magellan PRO software version 7.5.

Viral RNA was extracted from HEV RNA-positive plasma using the QIAsymphony automated system (QIAGEN, Hilden, Germany). A partial region of open reading frame 2 (ORF2) was amplified by nested reverse transcription-polymerase chain reaction (RT-PCR), as previously described (7), and positive PCR products were sequenced in both directions using the BigDye Terminator v3.1 Cycle Sequencing Kit on the SeqStudio Genetic Analyzer (Applied Biosystems, Waltham, MA, USA). After primer sequences were trimmed from both ends, a final 412-nt sequence was obtained and used for phylogenetic analysis.

The patient-derived sequence, named INMI 2527 CIBE and deposited in GenBank under accession number PZ471394, was edited and assembled using BioEdit v.7.2 and aligned with reference sequences using ClustalW implemented in MEGA 12 (27). For the patient-derived HEV sequence, the most similar sequences were retrieved from GenBank using BLASTn (https://blast.ncbi.nlm.nih.gov/Blast.cgi; accessed on 23 June 2026) searches against all HEV sequences available.

Genotype and subtype were assigned using the HEVnet typing tool (28).

Based on the genotype and subtype assignment obtained from BLASTn and HEVnet, two phylogenetic datasets were subsequently constructed using MEGA 12 (27). The first analysis was performed to confirm the placement of the patient-derived sequence within the assigned subtype and to assess its affiliation with the corresponding subclusters. The tree was inferred using the Maximum Likelihood method with the Tamura-Nei nucleotide substitution model (29), gamma-distributed rates and invariant sites. Branch support was assessed with 1,000 bootstrap replicates. The second analysis was performed to investigate the broader zoonotic and geographical context of the patient-derived strain using the closest BLASTn matches and selected human- and animal-derived HEV sequences. The second tree was generated using the Neighbor-Joining method (30) with the Tamura-Nei model (29), gamma-distributed rates among sites, and 1,000 bootstrap replicates. Appropriate reference sequences and outgroups were included in each analysis.

## 4 Virological, phylogenetic, and immune investigation

HEV RNA was detected in plasma two days after hospital admission, with a viral load of 15,002 IU/mL, whereas follow-up plasma samples collected at 3 and 6 months were HEV RNA-negative (**Figure 1**).

At hospital admission, the plasma IL-6 level was 9.12 pg/mL in the presence of detectable HEV RNA (15,002 IU/mL). This value was similar to those observed in patients with acute HEV infection [median: 4.36 pg/mL; interquartile range (IQR): 2.16–10.00 pg/mL] but higher than that reported in healthy donors [median: 1.31 pg/mL; IQR: 0.69–2.80 pg/mL]. In the follow-up sample collected after resolution of the purpura and clearance of plasma HEV RNA (3 months after admission), the IL-6 concentration exceeded the assay’s upper limit of quantification (>10 pg/mL).

BLASTn (**Supplementary Table 2**) and the HEVnet typing tool (**Supplementary Table 3**) concordantly assigned the patient-derived sequence to HEV genotype 3, subtype 3f. Following this genotype and subtype assignment, a confirmatory phylogenetic dataset was constructed. It included the patient-derived sequence, representative HEV-3f, HEV-3f1, HEV-3f2, and HEV-3f3 sequences, selected HEV-3c and HEV-3e reference sequences (FJ705359 and AB248521, respectively), and the HEV-4 sequence AB369690 as an outgroup (31). The Maximum Likelihood analysis confirmed the placement of the patient-derived sequence within subtype 3f and, more specifically, within the 3f2 subcluster (**Figure 2**).

**Figure 2:**
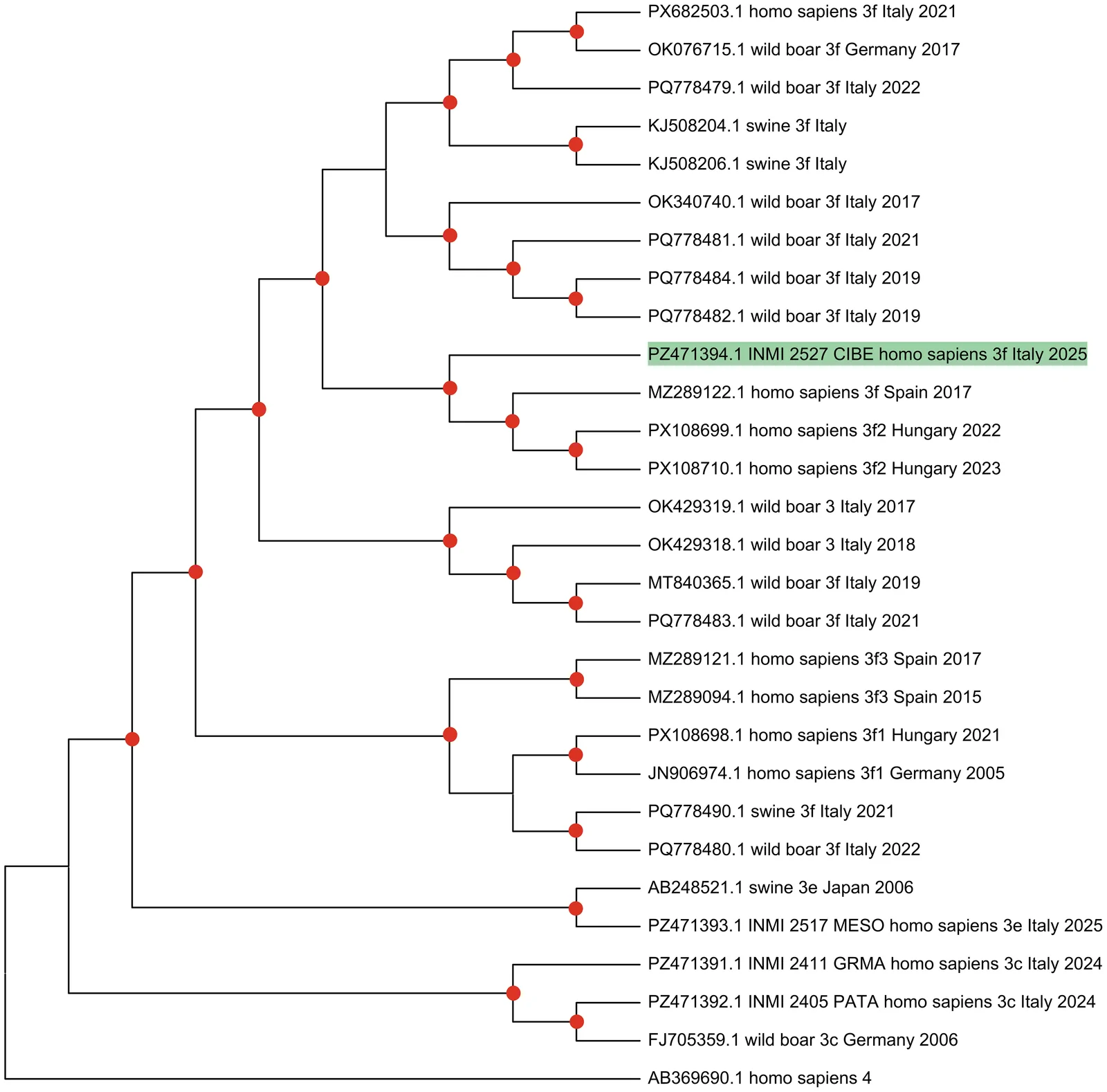
Phylogenetic analysis of the patient-derived HEV strain within subtype 3f and the 3f2 subcluster. Phylogenetic tree based on the partial ORF2 sequence, including the patient-derived strain, representative HEV-3f, HEV-3f1, HEV-3f2, and HEV-3f3 sequences, selected HEV-3c and HEV-3e reference sequences, and the HEV-4 sequence used as an outgroup. The patient-derived sequence is underlined in green. Bootstrap values ≥70 are indicated by red dots.

The closest human sequences in the tree were European HEV-3f/3f2 strains, including MZ289122.1 from Spain and PX108699.1/PX108710.1 from Hungary. BLASTn and pairwise identity analysis confirmed 95% nucleotide identity with these human sequences (**Supplementary Table 2**). Swine HEV-3f sequences from Poland showed 97% and 96% nucleotide identity. Among Italian human sequences, MN537876, isolated from a patient during a 2019 HEV outbreak (7), showed 91% identity with the CIBE sequence. In addition, the UM12 strain (Acc. No. OR795728) isolated in 2023 from a patient living in Rome who reported consumption of undercooked sausages, with possible inclusion of wild-boar meat, showed 90.02% identity with the CIBE sequence.

A second phylogenetic dataset was constructed to investigate the broader zoonotic and geographical context of the strain. It included the closest BLASTn matches and selected human, swine, and wild-boar HEV-3 sequences from Italy and other European countries. The HEV-3c sequence FJ705359 was used as an outgroup. In this analysis, the patient-derived sequence remained within the HEV-3f/3f2 diversity together with human, swine, and wild-boar sequences (**Figure 3**).

**Figure 3:**
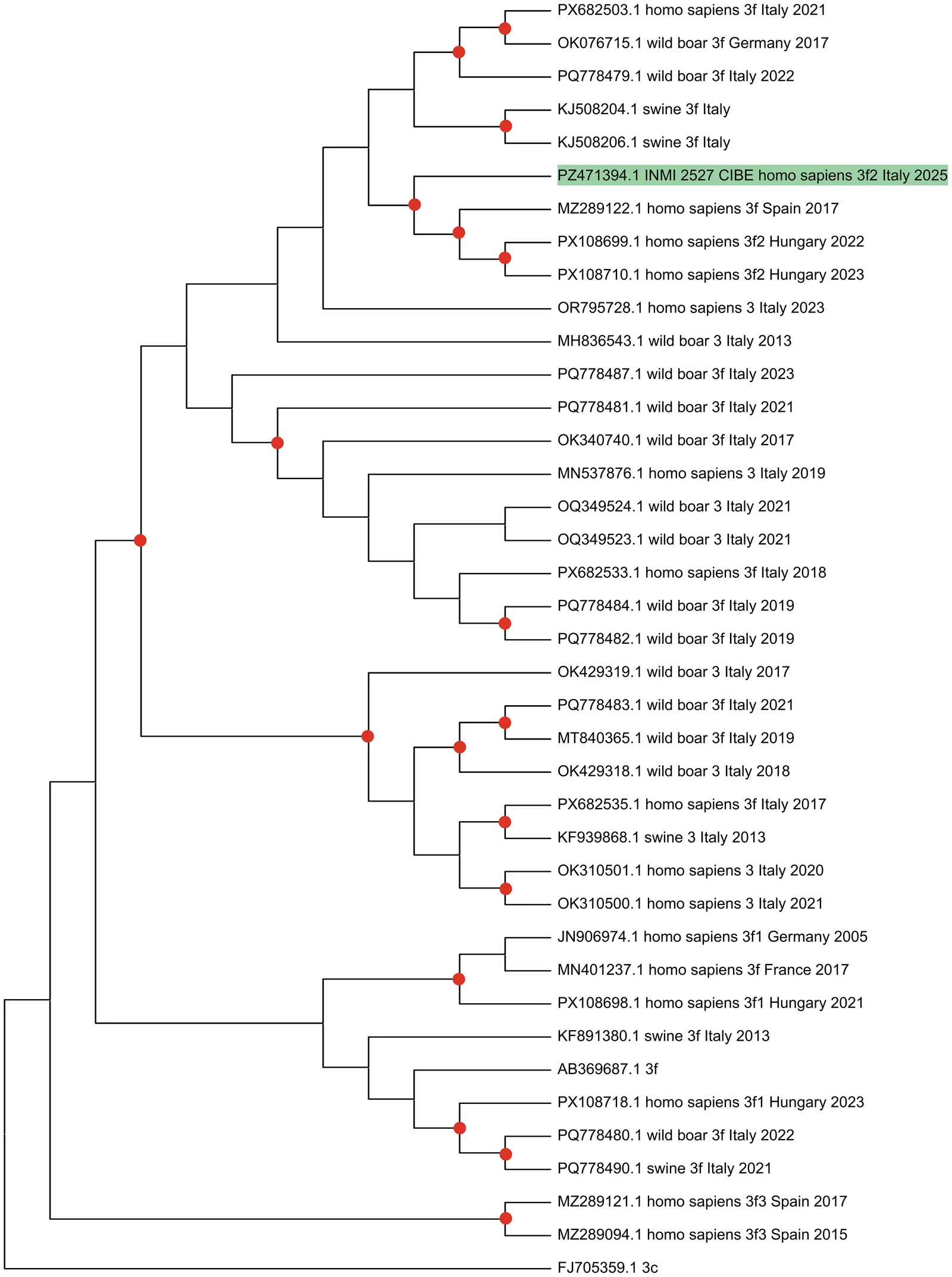
Phylogenetic analysis of the patient-derived HEV-3f strain with human, swine, and wild-boar sequences from Italy and Europe. Phylogenetic tree showing the broader HEV-3f zoonotic context, including the closest BLASTn hits and selected human, swine, and wild-boar HEV-3 sequences from Italy and Europe, and the HEV-3c sequence used as an outgroup. The patient-derived sequence is underlined in green. Bootstrap values ≥70 are indicated by red dots.

Italian animal-derived sequences, including swine sequences and wild-boar strains from Lazio, Umbria, and Abruzzo, showed approximately 91% nucleotide identity with the CIBE sequence. Although none of the available animal sequences was close enough to indicate a direct source of infection, their presence within the same HEV-3f-related diversity supports the zoonotic background of the strain. In this context, the patient’s reported consumption of raw or undercooked wild-boar sausage or meat in the month preceding symptom onset makes food-borne zoonotic transmission the most plausible route of infection.

## 5 Discussion and conclusion

The case described here highlights several aspects of the differential diagnosis of HSP-like purpura. Although this clinical manifestation appears to result from an immune-complex reaction to various antigenic stimuli, our understanding of the exogenous and host genetic factors contributing to this condition remains incomplete despite recent progress. The antigenic stimulus may derive from insect bites, food reactions, exposure to cold, drug allergies, bacterial infections such as Mycoplasma, Campylobacter, and Vibrio cholerae, or viral infections such as Parvovirus, Epstein–Barr virus, Adenovirus, Varicella virus, and yellow fever virus (32). Viral hepatitis A and B have also been reported as possible triggers of HSP onset (33–35). There is no specific laboratory test for this disorder, although elevated serum IgA levels are suggestive. However, the autoantigen to which IgA1 binds remains unknown (36). It has been hypothesized that microorganisms may share antigenic structures with human vessel walls (37). However, definitive data on this molecular similarity among the different pathogens associated with palpable purpura are not available.

HEV-associated HSP has been described in only one paediatric case, involving a six-year-old girl with acute HEV infection; however, HEV RNA detection, viral genotype, and subtype characterization were not reported (25). Our patient was a 91-year-old adult man who had never experienced allergic reactions to drugs and showed no signs of insect bites. The clinical and virological findings were consistent with acute HEV infection, including HEV IgM positivity and detectable HEV RNA. The purpura appeared concomitantly with the peak of transaminase levels and HEV viremia.

Further supporting the hypothesis that HEV may cause palpable purpura is the observation that HSP appears to have a seasonal predilection, with a peak incidence during the winter months, whereas occurrence during the summer months is relatively rare (38). During the same period, a peak in cases of acute hepatitis E has also been reported in Italy (39). From a clinical point of view, this case of acute hepatitis E did not differ from other acute hepatitis cases described in central Italy and associated with genotype 3, subtypes c, e, and f (40).

The HEV infection described here was caused by an HEV-3f strain belonging to the 3f2 subcluster. To our knowledge, this subtype/subcluster has not previously been associated with HSP-like purpura. HEV-3f has been reported in association with renal extrahepatic manifestations, including glomerulonephritis and mixed cryoglobulinemia (21, 41, 42); however, sequence comparison with these cases was not possible because GenBank accession numbers were not provided.

In our analysis, the CIBE strain showed high sequence similarity with European HEV-3f/3f2 strains, including human sequences from Spain (43) and Hungary (44) with 95% identity and Polish swine strains with 96–97% identity; Italian wild-boar strains from regions geographically close to the patient’s area of residence showed 91% identity. In addition, the patient-derived sequence showed 90.02% identity with the Italian human UM12 strain (Acc. No. OR795728) isolated in 2023 from a patient living in Rome who reported consumption of undercooked sausages, possibly containing wild-boar meat.

Although HEV-3f is the predominant subtype in Spain, HEV-3f-A1/3f1 is the main subcluster, whereas HEV-3f-B/3f2 was reported as rare (43). Therefore, the case described here expands the available information on HEV-3f/3f2 strains circulating in Europe.

To date, cutaneous manifestations associated with HEV have rarely been reported, and HEV-related HSP has been described in only one case, without detailed characterization of the viral subtype. Moreover, information on viral mutations potentially associated with extrahepatic manifestations remains limited. The L1110M polymorphism, described as characteristic of HEV-3e and HEV-3f-A2/3f3, has been proposed as a possible marker to be further investigated in relation to clinical severity (45). For these reasons, full-genome studies in larger case series are needed to clarify whether specific mutations or genetic patterns of HEV-3f may contribute to the induction of extrahepatic manifestations, including HSP-like purpura.

IL-6 has been implicated in the inflammatory activity of several vasculitic disorders. In ANCA-associated vasculitis (AAV), serum IL-6 levels have been reported to be increased during active disease and to correlate with selected clinical outcomes, including granulomatous manifestations and severe relapse in rituximab-treated patients (46). In our case, plasma IL-6 levels were 9.12 pg/mL at admission and >10 pg/mL at follow-up, after resolution of the purpura and clearance of plasma HEV RNA. Although IL-6 was measured in a single patient at only two time points, the observed concentrations were consistent with a systemic inflammatory response in the context of acute HEV infection and may reflect an inflammatory milieu related to the purpuric manifestation. Furthermore, the histopathological findings do not provide sufficient evidence to support a diagnosis of vasculitis, and an isolated elevation of serum IgA levels is not diagnostic of IgA vasculitis. Finally, the possibility that MGUS may have contributed to the development of the palpable purpuric eruption cannot be excluded.

In conclusion, data on viral factors potentially associated with extrahepatic inflammatory manifestations of HEV genotype 3, particularly subtype 3f, remain scarce. In this case, the temporal association between acute HEV infection and the palpable purpuric eruption suggests that HEV may have acted as a trigger, although a causal relationship cannot be established. This observation highlights the importance of considering both serological and molecular HEV testing in the differential diagnosis of patients presenting with palpable purpura or HSP-like manifestations. Full-genome analyses of larger case series will be essential to clarify whether specific HEV variants are associated with the development of extrahepatic inflammatory manifestations.

## Data Availability

The datasets presented in this study can be found in online repositories. The names of the repository/repositories and accession number(s) can be found below: https://www.ncbi.nlm.nih.gov/genbank/, PZ471394.
