# Supplementary material for: Case Report: Henoch–Schönlein-like purpura as an unusual presentation of acute hepatitis E virus infection
Source: Front Immunol. 2026 Oct 8;17:1937756. doi: 10.3389/fimmu.2026.1937756 (PMC13687430; doi:10.3389/fimmu.2026.1937756)
Supplement: Supplementary file 1 [file Table1.docx]

Supplementary Material

**Supplementary Table 1. Serial hematological and coagulation parameters during hospitalization.**

| Day after symptom onset | Platelet count (×10^9^/L) | Hemoglobin (g/dL) | INR | PTT (s) |
| --- | --- | --- | --- | --- |
| 6 | - | - | 3.06 | - |
| 9 | 104 | 11.8 | 1.54 | 30.4 |
| 10 | 138 | 11.4 | 1.45 | 40.0 |
| 15 | 136 | 10.8 | - | - |
| 16 | - | - | 1.64 | 38.7 |
| 17 | 139 | 10.0 | - | - |
| 18 | 146 | 10.2 | 1.45 | 39.4 |
| 20 | 161 | 10.3 | - | - |
| 23 | 188 | 11.1 | - | - |

Reference ranges: platelet count, 150–450 × 10⁹/L; hemoglobin, 14.0–17.5 g/dL; PTT, 29–44 s.
Abbreviations: INR, international normalized ratio; PTT, partial thromboplastin time; -, value not available.

**Supplementary Table 2.** Top BLASTn hits for the patient-derived sequence PZ471394.1_INMI_CIBE.

| GenBank Accession Number | Species | Country | Provence/Region | Years | Genome/subtype | Identities (%) |
| --- | --- | --- | --- | --- | --- | --- |
| MZ289122.1 | Homo sapiens | Spain |  | 2017 | 3f | 393/412(95%) |
| PX108710.1 | Homo sapiens | Hungary |  | 2023 | 3f2 | 392/412(95%) |
| PX108699.1 | Homo sapiens | Hungary |  | 2022 | 3f2 | 392/412(95%) |
| OQ686851.1 | swine | Poland |  | 2021 | 3f | 361/372(97%) |
| OQ686839.1 | swine | Poland |  | 2021 | 3f | 356/372(96%) |
| OQ349524.1 | wild boar | Italy | Umbria | 2021 | 3 | 375/410(91%) |
| OQ349554.1 | wild boar | Italy | Umbria | 2021 | 3 | 375/410(91%) |
| OK340740.1 | wild boar | Italy | Viterbo | 2017 | 3 | 376/412(91%) |
| OK310501.1 | Homo sapiens | Italy |  | 2020 | 3 | 376/412(91%) |
| PX682533.1 | Homo sapiens | Italy |  | 2018 | 3f | 376/412(91%) |
| KF939868.1 | swine | Italy |  | 2013 | 3 | 376/412(91%) |
| [MN537876.1](https://www.ncbi.nlm.nih.gov/nucleotide/MN537876.1?report=genbank&log$=nuclalign&blast_rank=22&RID=1FRD9VDE014) | Homo sapiens | Italy |  | 2019 | 3 | 376/412(91%) |
| OQ349523.1 | wild boar | Italy | Umbria | 2021 | 3 | 374/410(91%) |
| OQ349525.1 | wild boar | Italy | Umbria | 2021 | 3 | 374/410(91%) |
| OQ349527.1 | wild boar | Italy | Umbria | 2021 | 3 | 374/410(91%) |
| OK310500.1 | Homo sapiens | Italy |  | 2021 | 3 | 374/412(91%) |
| KJ508204.1 | swine | Italy |  | 2013 | 3 | 374/412(91%) |
| MN509471.1 | Homo sapiens | Italy |  | 2012 | 3 | 374/412(91%) |
| MH836543.1 | wild boar | Italy | Viterbo | 2013 | 3 | 373/411(91%) |
| KF891380.1 | swine | Italy |  | 2013 | 3f | 373/412(91%) |
| PX108732.1 | Homo sapiens | Hungary |  | 2025 | 3f2 | 373/412(91%) |
| PQ778487.1 | wild boar | Italy | Teramo | 2023 | 3f | 373/412(91%) |
| PX682535.1 | Homo sapiens | Italy |  | 2017 | 3f | 372/412(90%) |
| OR795728.1 | Homo sapiens | Italy |  | 2023 | 3 | 370/411(90%) |

**Supplementary Table 3.** Results from the HEVnet typing tool for patient sequence PZ471394.1_INMI_CIBE

| name | begin | length | end | BLAST result | Blast score | Ref seq | Reverse complement |  |
| --- | --- | --- | --- | --- | --- | --- | --- | --- |
| PZ471394.1_INMI_CIBE | 5919 | 412 | 6331 | Hepeviridae Paslahepevirus | 84.67153 | NC_001434 | false |  |
|  | Phylo-major result | Phylo-major support | Phylo-minor result | Phylo-minor support | Phylo-major best | Phylo-major best support | Phylo-minor best | Phylo-minor best support |
|  | HEV3 | 98.0 | HEV3f2 | 98.0 | HEV3 | 98.0 | HEV3f2 | 98.0 |
